# Efficacy of instillation of MB49 cells and thermoreversible polymeric gel in urothelial bladder carcinoma immunization

**DOI:** 10.1186/s42826-022-00122-7

**Published:** 2022-05-05

**Authors:** Jhonne Pedro Pedott Santana, Priscyla Daniely Marcato, Tais Nader Chrysostomo Massaro, Naiane Lima Godoy, Fernanda de Freitas Anibal, Ricardo Carneiro Borra

**Affiliations:** 1 Department of Genetics and Evolution, Federal University of Sao Carlos, São Carlos, Brazil; 2 GNanoBio, School of Pharmaceutical Science of Ribeirão Preto, University of São Paulo, Ribeirão Preto, Brazil

**Keywords:** Bladder cancer, Immunization, Immunogenicity, Chitosan

## Abstract

**Background:**

Activating the immune system for therapeutic benefit has long been a goal in immunology, especially in cancer treatment, but the low immunogenicity of antitumor vaccines remains a limiting factor in the fight against malignant neoplasms. The increase in the immunogenicity of weak antigens using biodegradable polymers, such as chitosan, has been observed in the field of cancer immunotherapy. However, the effects of the vaccine using a combination of tumor cells and a thermoreversible delivery system based on chitosan in bladder cancer models, mainly using the intravesical route to stimulate the antitumor immune response, are unknown. We propose to evaluate the efficacy of a polymeric gel matrix (TPG) formed by poloxamer 407 and chitosan, associated with MB49 cells, as an intravesical antitumor vaccine using a C57BL/6 murine model of bladder urothelial carcinoma. The effectiveness of immunization was analyzed with the formation of three experimental groups: Control, TPG and TPG + MB49. In the vaccination phase, the TPG + MB49 group underwent a traumatic injury to the bladder wall with immediate intravesical instillation of the vaccine compound containing MB49 cells embedded in TPG. The TPG group was subjected to the same procedures using the compound containing the gel diluted in medium, and the control group using only the medium. After 21 days, the animals were challenged with tumor induction.

**Results:**

In vitro tests showed loss of viability and inability to proliferate after exposure to TPG. In vivo tests showed that animals previously immunized with TPG + MB49 had higher cumulative survival, as well as significantly lower bladder weight and size in contrast to the other two groups that did not show a statistically different tumor evolution. In addition, the splenocytes of these animals also showed a higher rate of antitumor cytotoxicity in relation to the TPG and control groups.

**Conclusions:**

We can conclude that MB49 cells embedded in a polymeric thermoreversible gel matrix with chitosan used in the form of an intravesical vaccine are able to stimulate the immune response and affect the development of the bladder tumor in an orthotopic and syngeneic C57BL/6 murine model.

## Background

Urothelial bladder cancer (UBC) remains one of the most common types of malignant neoplasms in the world, especially in developed countries [1]. UBC is usually treated with chemotherapy, radiotherapy, BCG-based immunotherapy and surgery, used exclusively or in combination. However, these therapeutic modalities still cannot resolve most cases. The development of antitumor vaccines for treatment has gained priority in recent decades, but to date, the success rate has not reached the desired degrees [2, 3]. One of the reasons for this problem is associated with the fact that tumor antigens are recognized as tolerogens by the immune system due to their autogenous origin, mainly in the bladder environment [4, 5]. To overcome the tolerance barrier, researchers have been developing tumor cells (MB49) that express, isolated or in combination, cytokine genes that stimulate the immune response, such as: IL-2, GM-CSF, IL-21 [6, 7], and more effective vaccine adjuvants, such as: agonistic and non-agonistic toll-like receptors (TLRs) ligands, C-type lectin receptors (CLRs), retinoic acid inducible gene-I (RIG-I) receptors and stimulator of interferon genes (STINGs) [8].

Usually, the effectiveness of treatments with genetically modified cells has been evaluated using heterotopic syngeneic tumor models and intralesional treatment with genetically modified cells inactivated by radiation [6, 7]. Regarding the research that showed inhibition of tumor development, there is a question about the extent to which the results of this induction/stimulation model can be seen in models closer to reality, such as orthotopic syngeneic tumor models.

On the other hand, the choice of the immunization route is a key factor in directing effector immune cells to the target mucosa. Comparing the intravaginal (IVAG), intranasal (NAS) and subcutaneous (SC) routes, Domingos-Pereira et al. [9] showed that the SC and IVAG routes were the most effective in inducing antigen-specific CD8 T cells in the bladder. However, after SC immunization, the regression of tumors located in the bladder was lower than the regression of tumors in the genital mucosa, suggesting that the bladder has characteristics of an immunotolerant environment [10].

Thus, we can consider that the direct stimulation of the immune response on the urothelium through intravesical (IVES) treatment, as after a transurethral resection procedure, could be as effective or more effective than immunization by other routes of administration. In this case, the induction of a local response associated with a local inflammatory process, triggered by the surgical procedure itself, could disrupt bladder immune tolerance and potentialize the antitumor immune response [5, 11]. However, the physiological characteristics of the bladder generate additional challenges for the introduction and maintenance of antigens in the vesicle for as long as necessary for the activation of an anti-tumor immune response. For this purpose, the vaccine compound should have the ability to remain adhered to the epithelium or connective tissue and breach the blood-urine barrier in order for the dendritic cells to reach the site to capture and process the antigens before their elimination by the urinary flow. The use of biocompatible gels with mucoadhesive properties could be used to solve this difficulty.

Among the widely studied polymers, chitosan stands out for being a natural cationic polymer of low cost, with high biocompatibility and versatility in different formulations [12]. Chitosan has positive charges that interact electrostatically with negative sialic acid residues, glycosaminoglycan, and mucin molecules present on the mucosal surface, promoting mucoadhesiveness. It also has the property of aiding in the antigens absorption since it is capable of increasing paracellular permeability through the opening of epithelial junctions probably caused by the electrostatic interaction with the alpha-v beta-3 (α_V_β_3_), and the promotion of desquamation and necrosis of the urothelium superficial cells [13, 14]. Thus, the use of chitosan has been explored in different delivery systems, allowing chemotherapy or immunomodulators such as IL-12 to accumulate and stay longer in contact with the urothelium [15]. Furthermore, chitosan exhibits properties that characterize it as an excellent immunological adjuvant. A viscous chitosan solution is capable of creating antigen deposit and inducing transient cell expansion in draining lymph nodes, increasing antibody titers and splenic proliferation of antigen-specific CD4 T cells similarly to incomplete Freund's adjuvant (IFA) and superior to aluminum hydroxide [16]. In addition, chitosan can be combined with poloxamer 407 to obtain a thermoreversible gel and increase the viscosity and adherence of the compounds to the mucosa [17]

Poloxamer gels exhibit an interesting property of reversible thermal gelation showing low viscosity solutions at room temperature and change to gel at body temperature. This gel is an attractive formulation thermosensitive for topical delivery, in the special for the intravesical route, due to its facility to the administration at low temperature (~ 25 °C) and gel formation inside the bladder at body temperature. Furthermore, poloxamer is biocompatible and has low toxicity [18–21] Westerink et al*.* (2002) verified that the thermosensitive gel poloxamer F127/chitosan showed a significantly enhance the immune response in the intranasal tetanus toxoid administration [22]. Thus, this type of gel of poloxamer/chitosan could be used to advantage as an immunotherapeutic delivering vehicle to the urothelium surface.

**Fig. 1:**
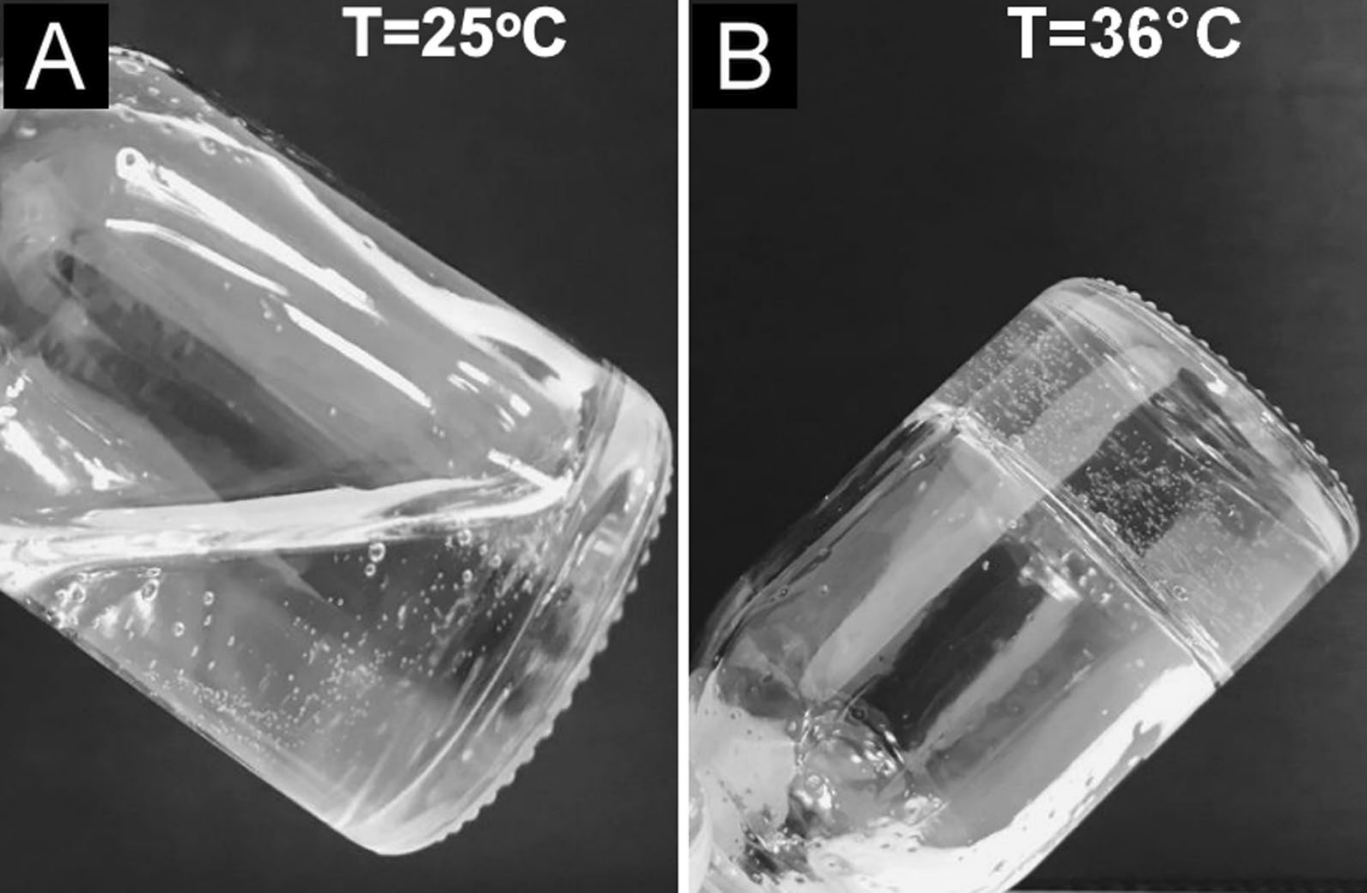
Thermoreversible polymeric gel (TPG) liquefies **A** at room temperature, 25 °C, and gels at **B** body temperature, 36 °C

Research involving the investigation of new techniques that explore intravesical immunotherapy could be of great relevance for the treatment of bladder cancer. Therefore, the aim of the present study was to evaluate the efficacy of a thermoreversible polymeric gel matrix composed of chitosan and poloxamer 407 (TPG, Fig. 1) as an intravesical vehicle and adjuvant for tumor antigens. For this purpose, the orthotopic syngeneic tumor model was used and the antitumor response was evaluated after immunization with MB49 cells embedded in TPG matrix.

## Results

### Effect of TPG concentration on MB49 cells viability

**Fig. 2:**
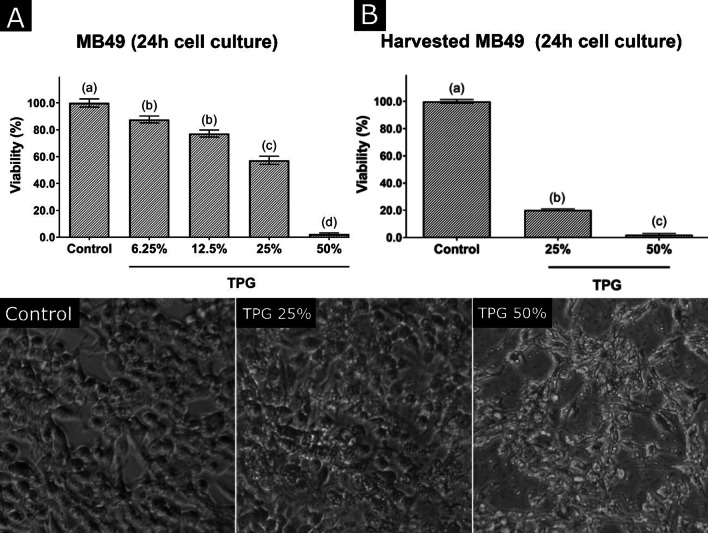
**A** Cell viability quantified by the resazurin assay of MB49 cells exposed for 24 h to different concentrations of TPG (6.25, 12.5, 25 and 50%). Cells grown in the absence of TPG were considered as a control. **B** Viability of harvested MB49 cells initially exposed to TPG (25 and 50%) for 24 h and re-plated for an additional 24 h. Cells grown in the absence of TPG were considered as a control. Data are expressed as mean ± SEM and values with *p* < 0.05 were considered significant after analysis with the one-way ANOVA test and Tukey`s test for post-hoc analysis. Bars with distinct letters show significant differences in viability between groups. Micrographs of the MB49 culture with and without TPG exposure (25 and 50%), showing that despite the cell viability having significantly decreased, especially in the TPG50 group, the cells remained adhered to the microplate

The viability of MB49 embedded in 25 or 50% of TPG show a significant reduction in the viability of MB49 cells after TPG exposure at concentrations of 50% and 25% in comparison with the control group without TPG (Fig. 2A). Figure 2 Micrographs shows that, even after loss of viability, cells continue to adhere to the three-dimensional matrix promoted by the application of TPG50. To confirm whether cells trapped on TPG were not viable, a new resazurin viability assay was performed, this time with the gathering of adherent cells by trypsinization after exposure to TPG at 25 and 50%. The results again showed a significant reduction in cell viability after exposure to TPG (Fig. 2B).

**Fig. 3:**
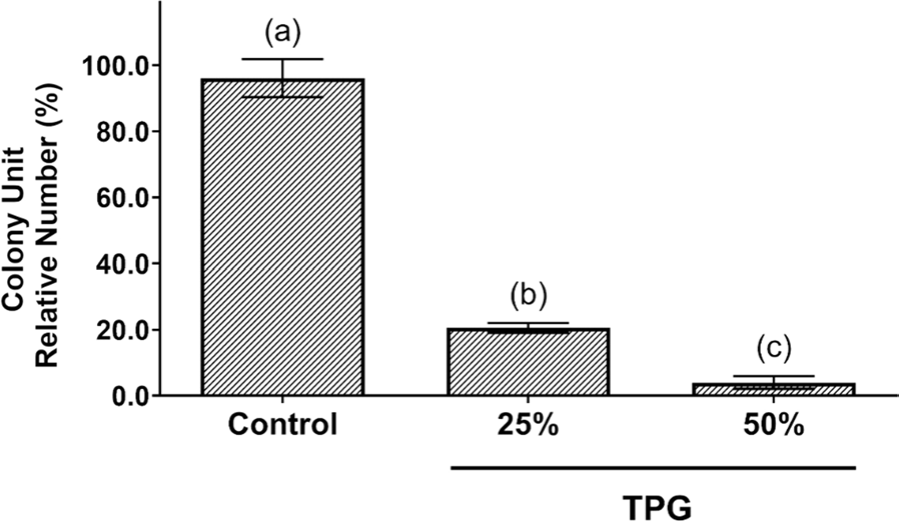
Evaluation of MB49 cell colony formation after exposure to TPG (25 and 50%) for 24 h, followed by replating with DMEM for 48 h. Cells grown in the absence of TPG were considered a control. Data are expressed as mean ± SEM and values with *p* < 0.05 were considered significant after analysis with the one-way ANOVA test and Tukey`s test for post-hoc analysis. Bars with distinct letters show significant differences in their ability to form colonies

In order to assess the residual capacity of proliferation and colony formation of MB49 tumor cells, after exposure to TPG at 25 or 50% for 24 h, a clonogenic assay was performed. After the exposure of MB49 tumor cells to TPG for 24 h, it was observed that in the group exposed to the lowest concentration of TPG investigated (25%), 20% of the cells maintained the capacity to proliferate. When exposed to a higher concentration of TPG (50%), only 4% of recovered cells were able to proliferate and form colonies (Fig. 3).

### TPG50 interference in the intravesical tumor model development

Based on the absence of viability of MB49 cells, a concentration of 50% TPG (TPG50) was chosen to evaluate the possibility of tumoral implantation. To compare the rate of tumor development, 3 groups of C57BL/6 female mice were used: group A1 received an intravesical inoculation of MB49 cells embedded in TPG (TPG50); group A2 received only the MB49 cells; and group A3 received only the inoculation of TPG (TPG50) to observe possible side effects promoted by the gel on the animals.

**Fig. 4:**
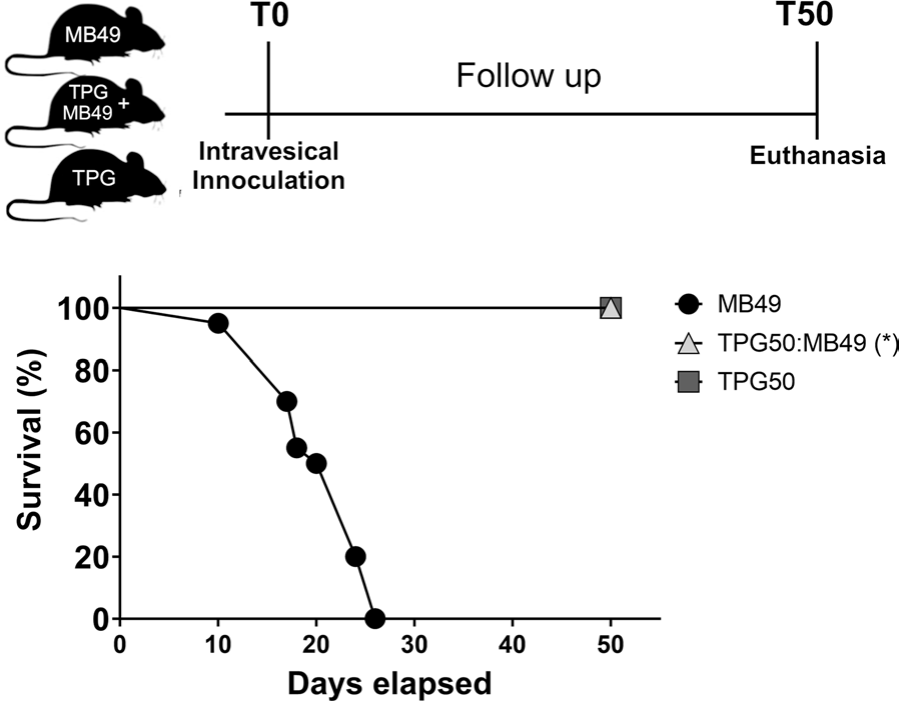
Cumulative survival of animals after inoculation of MB49 cells in the presence (Group A1, TPG50:MB49) or absence (Group A2, MB49) of TPG. In order to observe possible side effects promoted by TPG, a group was also created in which only TPG was inoculated in the animals (Group A3, TPG50). The animals were followed for 50 days, in which the appearance of signs corresponding to tumor development was observed. Significant survival was obtained 26 days after death or euthanasia of all animals in the Group A2, in which only MB49 cells were inoculated, by the Kaplan Mier method associated with the Gehan-Breslow-Wilcoxon test (*p* < 0.005). Asterisk denotes statistical significance compared to the control group (Group A1)

Cumulative survival curve showed that the tumor development model using the delivery system composed of chitosan and poloxamer 407 at the time of vesical instillation of MB49 cells, promoted a survival rate of 100% of the animals. After 26 days of follow-up, all animals in Group A2, inoculated only with MB49 cells, had already been euthanized or died due to the tumor. However, in this same follow-up period, none of the animals in Group A1 or A3, inoculated with MB49 cells or TPG, died as a result of the induction of the orthotopic syngeneic tumor model of bladder urothelial carcinoma. The follow-up of the animals in Group A3 showed that TPG did not cause any observable side effects in the animals (Fig. 4).

### Immunogenic effect of TPG50

**Fig. 5:**
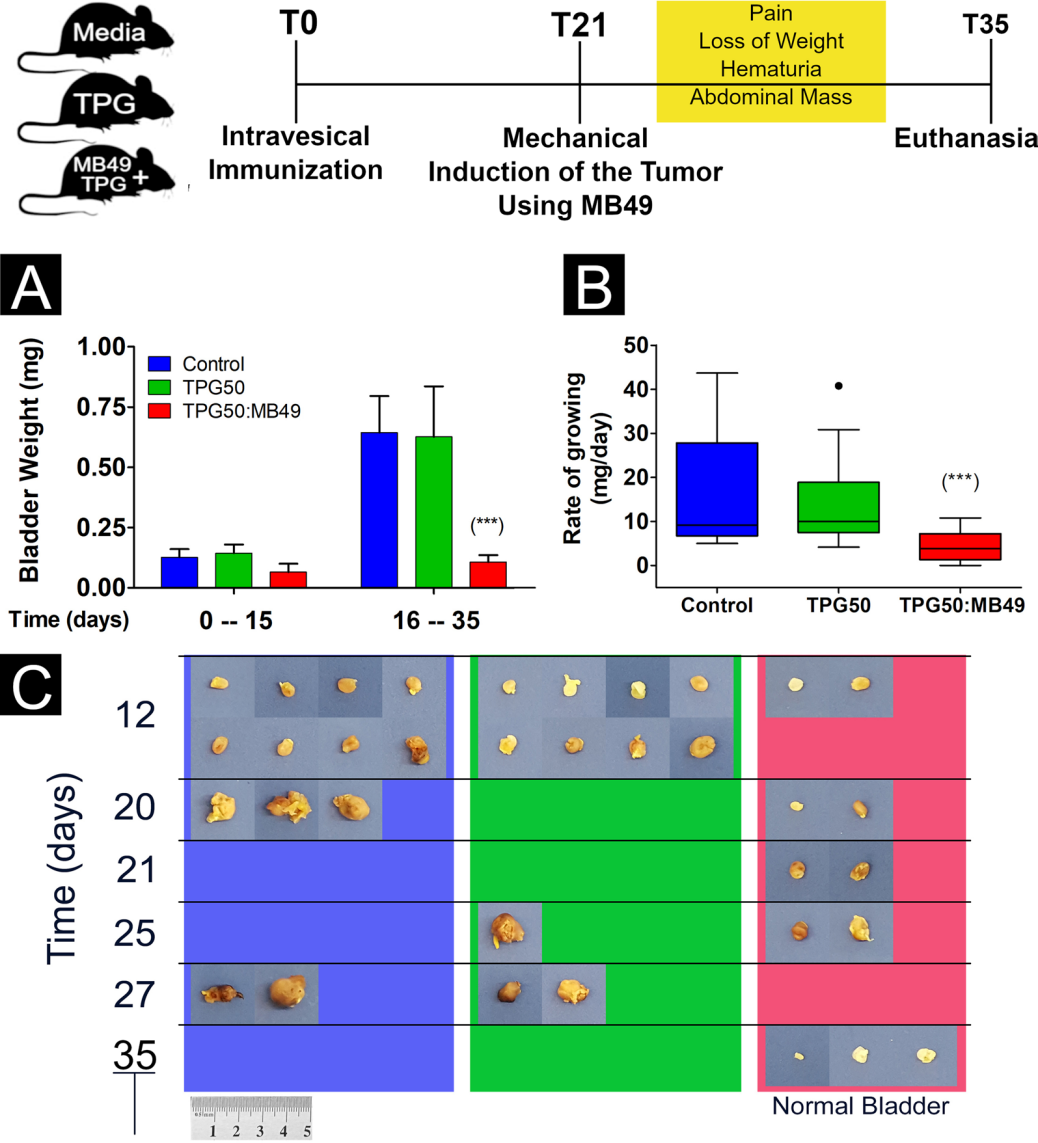
Evolution of tumor development of animals previously immunized with non-viable MB49 tumor cells and TPG (Group B1, TPG50:MB49) or not immunized (Groups B2, TPG50, and B3, Control). After 21 days, the immunization challenge with MB49 cells (1 × 10^5^ cells/mouse) was performed and the animals were followed up for 35 days. In case of signs of tumor development, the animals were euthanized and the bladders were removed and weighed. **A** Bar Graph showing mean ± SEM values of bladder weights. **B** Box Plot (Tukey`s test) showing the distribution of calculated tumor growth rates dividing the bladder weight by the value in days from induction to euthanasia of the animals. **C** Images of the animals' bladders after euthanasia, distributed according to the day of collection. Statistical analysis performed using the non-parametric Kruskal–Wallis test followed by the Dunn's multiple comparisons test, (***) *p* < 0.01 compared to the control group

The follow-up of the animals showed that in the group that had been previously immunized with TPG50:MB49, the final survival rate after 30 days was 20%. This rate was significantly higher in this group after comparing it to the non-immunized groups, in which there were no survivors after 30 days of follow-up (Fig. 5). Three animals from the group treated with TPG50 + MB49, euthanized at the end of the experiment, did not show tumors in the examined bladders. After bladder extraction from euthanized animals, it was possible to visually compare bladder size between groups. Analyzing Fig. 5, it is possible to observe that the bladders of the previously immunized group (TPG50:MB49) have statistically (*p* < 0.01) lower values for weight and tumor growth rate than bladders belonging to the other groups (Fig. 5).

### TPG50:MB49 immunization effect on the antitumor response of splenocytes

**Fig. 6:**
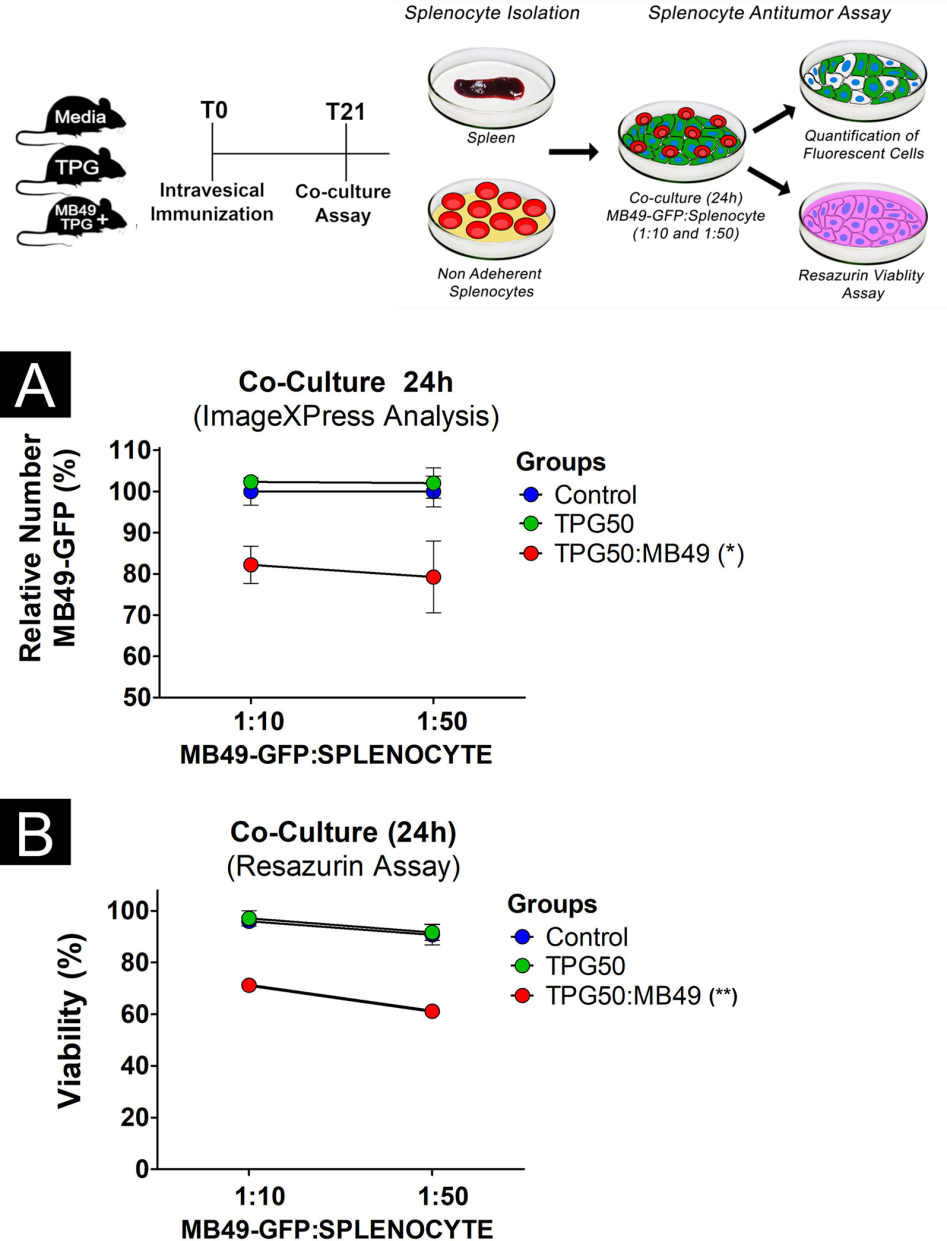
Evaluation of cytotoxicity by quantification of MB49:GFP^+^ cells death by fluorescence analysis (**A**) and by resazurin assay (**B**). Statistical analyses were performed using the two-way ANOVA test (*p* < 0.05). Statistical significance compared to the control group with *p* < 0.01 (**) and *p* < 0.05 (*)

In addition to the analysis of the vesical tissue, the antitumor immune response was evaluated by a co-culture assay with MB49:GFP^+^ (Fig. 6A) and purified splenocytes from the three groups. The results showed that the splenocytes from the TPG50:MB49 group significantly (*p* < 0.05) eliminated a greater amount of MB49:GFP^+^ cells in relation to the control and TGP50 groups in the two tested proportions of 1:10 and 1:50. In addition, the co-cultured cell viability of the three groups was quantified using the resazurin assay. The data show a significant (*p* < 0.01) loss of viability in the co-cultivation associated with the TPG50:MB49 group, mainly in the 1:50 proportion (Fig. 6B). Between the TPG and control groups, there were no statistical differences in terms of MB49:GFP^+^ cell loss (Fig. 6A) or cell viability (Fig. 6B).

## Discussion

### MB49 cells viability after exposure to TPG

In our case, the ideal concentration of TPG to embed viable MB49 cells should be able to destroy cell viability while keeping the cell body trapped in the gel matrix, allowing tumor antigens to be released for a longer period of time without the risk of tumoral implanting into the tissue. To determine the concentration of work, cytoxicity and clonogenic assays were carried out to analyze the viability of MB49 embedded in 25 or 50% of TPG. The significant reduction in the viability of MB49 cells after TPG exposure were surprising since the TPG used is considered an inert and non-toxic delivery system [23]. However, this finding corroborates other works that show cytotoxic effects of chitosan on tumor cells [24, 25]. The study by Salehi et al. (2017) demonstrates the antiproliferative effect of chitosan on MDA-MB-231, MCF-7 and T47D cells lineages, promoting apoptosis in these breast cancer cells, in addition to demonstrating the lack of toxicity for cells normal fibroblasts (L929) [26]. The growth inhibitory effect after treatment with chitosan was also observed in human bladder cancer cell lines demonstrated by DNA fragmentation analysis in which there was the induction of apoptosis pathways leading to caspase 3 activation [27]. Thus, when comparing our results with those found in the literature, we can suggest that TPG may promotes programmed cell death in tumor cells.

The fact that MB49 cells exposed to 50% TPG with viability below 2% continue to adhere can be explained by the formation of a three-dimensional matrix promoted by the application of the TPG50. The morphological changes observed in this figure are related to the loss of cell viability and the presence of this three-dimensional matrix (Fig. 2 Micrographs). This matrix enfolds the cells and prevents them from releasing even after death, corroborating several tissue engineering studies which propose the creation of three-dimensional matrices from polymeric gels [28–30].

In the resazurin viability assay with the gathering of adherent cells by trypsinization after exposure to TPG (Fig. 2B), the harvested cells were replated for 24 h and, after this period, the supernatants were discarded and the cell viability assay by resazurin was performed. The viability rate of TPG50 group was approximately 2%, maintaining the same rate found in the previous experiment. In the TPG25 group exposed to 25% TPG, the viability rate was lower than that found in the previous experiment, probably due to cell separation procedures. This new cell viability assay confirmed that cells present in the TPG matrix and re-plated were not actually metabolizing resazurin as they were already non-viable.

For the clonogenic assay, cells were collected, re-plated (1 × 10^3^ cells/well) for 48 h, and colony-forming units (CFUs) were counted under an optical microscope (Fig. 3). We can observe that 20% of the cells maintained the capacity to proliferate when exposed to TPG25 and only 4% of recovered cells were able to proliferate and form colonies when exposed to TPG50. This result corroborates those found in the resazurin assays described above (Fig. 2), in which cell viability was also reduced after exposure to TPG, especially in the TPG50 group.

### Tumor progression after TPG50 exposure

The analysis of the cumulative survival curve showed the absence of tumor development after intravesical instillation of TPG50:MB49 could be associated with a combination of factors related to the imprisonment of cells in the TPG matrix, the loss of viability of MB49 cells and the development of an antitumor immune response (Fig. 4). This immunostimulant activity, together with the structural similarities between chitin derivatives and glucans, has led many researchers to investigate the adjuvant properties of chitosan [31–33].

### Immunogenic effect of TPG50

The investigation of the hypothesis of the potential immunogenic effect of thermoreversible polymeric gel with chitosan within the vesical environment began with the immunization and tumoral challenge of C57BL/6 mice. To perform this experiment, 3 groups of animals were submitted to immunization procedures with MB49 cells, TPG50, or TPG50:MB49 cells, after mechanical trauma of the bladder. The tumoral challenge with MB49 was performed 21 days after immunization, as the peak of cell-mediated immune response promotion after chitosan administration is between 14- and 21-days post-injection [16]. The appearance of signs corresponding to tumor development was observed more quickly in animals from the non-immunized groups, with animals showing hematuria or pain behavior on the 11th day after the challenge. The first animals were euthanized on the 12th day, when 8 animals from each non-immunized group (n = 16) and 2 animals from the immunized group were euthanized.

Tumor induced using 1 × 10^5^ cells/mice and traumatic bladder injury is considered to be highly aggressive, especially in relation to the induction models with chemical injury. By this protocol, the induction success rate is 100%. If the number of inoculated cells were smaller, the differences found for the immunized animals would probably also be greater. Another factor to be considered is the influence of the traumatic injury caused by the rotation of the catheter tip in the urinary bladder mucosa before the inoculation of the compounds. Mechanical trauma probably induces an inflammatory process, similarly to transurethral resection (TUR), which can help attract dendritic cells responsible for activating the anti-tumor immune response [34, 35]. However, the inflammatory process by itself, without the presence of tumor antigens, is not capable of inhibiting further tumor development. The animals belonging to the control group and the TPG group suffered the same trauma during the immunotherapeutic treatment, but they evolved in an unfavorable way. Not even the presence of TPG, when inoculated without MB49 cells, stimulated an antitumor response (Fig. 6). Probably, in this case, the isolated administration of TPG without antigen (TPG group) induced only a temporary non-specific innate immune response, incapable of inhibiting the tumor in the phase of tumoral challenge [31, 36]. Marcinkiewicz et al. (1991) showed that the intraperitoneally inoculation of chitosan alone in mice is able to increase the humoral immune response, but not the cell-mediated immune response [37].

The concentration and pH of the TPG compound used in immunization may also be a relevant factor in promoting the immunization of animals since it induces the death of a large part of the cells and creates a slow-release deposit of tumor antigens. The modulation of immunogenicity after the induction of cell death was described by Luo et al. (2019) in an experiment involving A529 cells lineage of lung cancer in which they compared the administration of a whole-cell vaccine with cells killed by irradiation with the administration of non-irradiated cells, confirming that cell death increased the immunogenicity of this type of vaccine, promoting an increase in the response of specific T cells against the tumor [38]. Chitosan is a promising vaccine adjuvant that has high biocompatibility and can retain antigens at the inoculation site for up to 7 days until metabolized [16, 39].

### Antitumor response of splenocytes

Co-culture analysis was performed using the ImageXpress® system where viable MB49:GFP^+^ can be identified by fluorescence and automatically quantified. The splenocyte is an organ with a high proportion of CD8 compared to CD4 T cells. The most likely scenario in an antitumoral co-culture assay utilizing splenocytes is that activated CD8 T cells are the major cells responsible for antitumoral activity [40, 41]. The assay with splenocytes collected from immunized mice was also used by Bauer et al. (2016) to demonstrate antitumor cytotoxicity against neuroblastoma cells (N2a) using flow cytometry [42]. The effects of the intravesical immunization on splenocytes could imply that stimulation was effective in eliciting a response in the local lymph nodes that drain the bladder as well as in activating resident mucosal immune cells.

The data from the above experiments show that it is possible to use a 50% concentration intravesical thermoreversible gel containing antigens to induce antitumor immunity and inhibit tumor growth. Inoculation of 100 μL of TGP50 is not toxic to mice, and even when mixed with viable MB49 cells and applied to mechanically traumatized mucosa, it does not induce bladder tumor development.

## Conclusions

MB49 tumor cells embedded in a thermoreversible polymeric gel matrix, composed of chitosan and poloxamer 407 and applied intravesically, are able to stimulate an antitumor immune response and inhibit the development of urothelial bladder carcinoma in a C57BL/6 orthotopic syngeneic murine tumor model.

## Methods

### Preparation of thermoreversible polymeric gel (TPG)

The delivery system proposed for intravesical inoculation together with MB49 cells consists of a binary polymeric system (Fig. 1) composed of poloxamer 407 (Sigma-Aldrich, St. Louis, USA) and 0.5% chitosan (medium molecular weight, 75–85% deacetylated, Sigma-Aldrich) in a 0.5% acetic acid solution. Chitosan (0.5–1.5% w/w) was initially dissolved in a solution of acetic acid (0.5% v/w), which improved mechanical and mucoadhesive properties that could provide prolonged retention time [17]. The chitosan solution was refrigerated and used as a solvent for the poloxamer (14–20% w/w) dispersion. All formulations had pH levels between 6.0 and 6.5 [17].

### MB49 tumor cell culture

Murine transitional carcinoma cell line (MB49-NCI Thesaurus Code: C25823), donated by Dr. Yi Lou (University of Iowa), were cultivated in Dulbecco’s Modified Eagle’s Medium (DMEM) with high glucose and 2 mmol/L of L-glutamine (Cultilab, Campinas, Brazil), supplemented with 10% fetal bovine serum (FBS, Cultilab) and 1% penicillin/streptomycin antibiotics (Vitrocell Embriolife, Campinas, Brazil) (complete DMEM). The cell culture was maintained in an incubator at 37 °C in an atmosphere of > 95% humidity and 5% carbon dioxide, in 75 cm2 bottles. Cell growth and adherence were monitored daily, under an inverted microscope, until 90% confluence of the cell monolayer was observed [43]. Adherent cells were then removed from the surface with the addition of a 2.5% trypsin solution (Life Technologies, Carlsbad, CA, USA) and direct cell quantification, in a Neubauer chamber, was performed with the aid of a Trypan blue solution. at 0.4% (Life Technologies), where cellular integrity was confirmed by the exclusion method.

### Effect of TPG concentration on the MB49 cells viability and clonogenicity

#### Resazurin viability assay

The biocompatibility of TPG with MB49 cells was evaluated by culturing, in quadruplicate and overnight, MB49 cells (1 × 10^5^ cells/well in 96-well microplates) and exposing them to 200 μl of a solution of complete DMEM and TPG at different concentrations (0 [negative control], 6.25 [TPG 6.25], 12.5 [TPG 12.5], 25 [TPG25], and 50% [TPG50]) for 24 h. After the period of incubation, the resulting cells were washed with phosphate-buffered saline (PBS 1X) and the cell viability test was performed by adding 20 µL/well of resazurin (70 µmol/L solution diluted in PBS 1X, Sigma-Aldrich, USA) to fresh DMEM without SFB. The absorbance was measured in a spectrophotometer after 3 h of metabolization of resazurin in resorufin using a spectrophotometer with wavelengths of 570 and 600 nm as reference, following the methodology described by Borra et al. (2009) [44]. The cell without treatment was used as a negative control with 100% viability and the cell treated with 1% triton X for 10 min before the quantification, as a reference to 0% viability.

To eliminate the gel interference in the viability measurement, a new assay with cell removal after TPG exposure was performed. In this new assay, MB49 cells were initially cultured overnight in 6-well microplates, until they reach 90% confluence, and exposed to solutions containing 25 and 50% TPG in complete DMEM for 24 h. Next, the adherent cells were removed from the surface of the wells by trypsinization (2.5%, Life Technologies, Carlsbad, USA), diluted in 5 mL of DMEM with 10% of FBS, centrifuged at 200 g, resuspended in 200 μL and re-plated in 96-well microplates for another 24 h. Following this time period, the viability was quantified using the same resazurin protocols as before.

#### Clonogenic assay

The influence of TPG on the proliferation capacity of tumor cells was investigated through the colony formation assay. Initially, 5 × 10^5^ MB49 cells were cultured in 6-well microplates with 5 mL of high-glucose DMEM medium complemented with 2 mmol/L of L-glutamine (Cultilab), 10% FCS (Cultilab), and 1% penicillin/streptomycin (Vitrocell Embriolife).The microplate was incubated for 48 h at 37 °C in a 5% carbon dioxide atmosphere. When the cell monolayer reached 90% confluence, the supernatant was discarded, and the cells were exposed for another 24 h to 5 mL of a solution of complete DMEM and TPG at different concentrations (25 and 50%). After that, adherent cells were collected from the surface of the wells using trypsin (2.5%), washed/neutralized with DMEM + 10% FBS, centrifuged at 200 g, and resuspended in 1 mL of DMEM. The cell quantification of the control group (without exposure to TPG) was performed with 0.4% Trypan blue (Life Technologies) using a Neubauer chamber.

The cells from the control group (0 percent TPG) were diluted in a volume of complete DMEM determined to be able to culture 1 × 10^3^ viable MB49 cells/well. Using 48-well microplates, the same volume was utilized to plate cells from the other TPG groups in quintuplicate. The microplates were kept in an incubator for 48 h until the colony-forming units were observed.

### TPG interference in the development of the intravesical tumor model

Based on the absence of viability of MB49 cells, the concentration of 50% TPG (TPG50) was chosen to evaluate tumor induction interference. To compare the rate of tumor development, 3 groups of C57BL/6 female mice from Biotério Central of the University of São Paulo-Ribeirão Preto (USP-RP) were used: Group A1 (n = 20), submitted to intravesical inoculation of a solution containing 50 μL of cell suspension with 5 × 10^5^ MB49 cells in DMEM and 50 μL of TPG (TPG50); Group A2 (n = 20), exposed to 100 μl of a solution containing 5 × 10^5^ MB49 cells in DMEM, without any delivery system; Group A3 (n = 10) submitted to intravesical inoculation of a solution containing 50 μL of DMEM and 50 μL of TPG (TPG50) without MB49 cells in order to observe possible side effects promoted by the gel to the animals. All procedures were approved by the Ethics Committee on Animal Use of The Federal University of São Carlos (CEUA/UFSCar, n◦ 9234221018 and 4556170619). Before induction procedures, mice were anesthetized intraperitoneally with ketamine (90 mg/kg, Dopalen®, Ceva, Paulínia, Brazil) and xylazine (10 mg/kg, Anasedan®, Ceva). Transurethral catheterization was performed with a needleless 24G polyethylene intravenous catheter (0.7 × 19 mm) lubricated with petroleum jelly (Vaseline®, Unilever, London, England). After emptying the bladder, a traumatic injury to the bladder epithelium was performed with a catheter needle with a curved tip (5 to 7 degrees), inserted into the catheter and rotated 5 times in order to promote tumor induction [45]. Catheterized animals were kept in the dorsal decubitus positioning for 45 min with the syringe attached to the catheter. Intravesical inoculations were performed randomly among the animals and, subsequently to the inoculation experiment, they were kept in separate boxes according to their group. Lesions were blindly induced in the animals by an operator who had no idea which group the animals belonged to. The tumor induction rate was determined by following the evolution of the lesions for up to 50 days. During this period, the appearance of indicative signs of tumor presence, such as pain behavior, hematuria, palpable abdominal mass or recurrent weight loss were evaluated. After 50 days, all remaining animals were euthanized by an overdose of chemical anesthetics (ketamine, 270 mg/kg, and xylazine, 30 mg/kg) and the bladders were examined for signs of tumor damage.

### Immunogenic effect evaluation of the TPG50:MB49

#### Mice immunization

Based on the absence of tumor induction, the 50% concentration of TPG (TPG50) was chosen to serve as an adjuvant in the immunization with non-viable MB49 cells. For this, 54 mice were randomly divided (www.random.org) into three groups: Group B1 (n = 18), submitted to intravesical inoculation of a solution containing 50 μL of cell suspension with 1 × 10^5^ MB49 cells in DMEM and 50 μL of TPG (TPG50); Group B2 (n = 18), submitted to intravesical inoculation of a solution containing 50 μL of DMEM and 50 μL of TPG (TPG50), without MB49 cells; Group B3 (n = 18), exposed to 100 μl of a solution containing only DMEM without any delivery system or tumor cells. Before exposure, the animals were anesthetized and a traumatic injury to the bladder epithelium was performed according to the methodology described above.

#### Tumoral challenge

Twenty one days after the initial inoculation, three animals from each group were separated to collect the splenocytes and the rest (n = 15) were submitted to the induction procedures of the orthotopic syngeneic tumor model of urothelial bladder cancer, in which a solution containing 1 × 10^5^ MB49 cells in DMEM were inoculated into the bladder after anesthesia and traumatic injury to the bladder epithelium.

#### Effect of TPG50:MB49 immunization on tumor development

To determine post-challenge tumor development, the animals were kept under daily observation for up to 35 days. Tumor development was assessed through the appearance of indicative signs of tumor presence such as pain behaviors, hematuria, increase in abdominal volume, or recurrent weight loss. As soon as the animals showed any of the indicative signs, they were euthanized and the bladders were removed to quantify the tumor mass, thus avoiding unnecessary suffering to the animals. After euthanasia, the abdominal cavity, liver, spleen, and kidneys were examined looking for metastases. Before the bladder was removed, it was examined for signs of a tear in the muscle layer. The difference between tumor tissue and normal bladder tissue is often easy to identify since the tumor mass is well vascularized. The collected bladder tissue samples were washed in 0.9% saline solution, dried and weighed. Tumor growth rate values were calculated by dividing the bladder weight by the value in days from induction to euthanasia of the animals.

### TPG50:MB49 immunization effect on the antitumor response of splenocytes

#### Isolation of murine splenocytes

The splenic tissues of three animals from groups B1, B2 and B3 (n = 9), which did not undergo tumor induction, were collected. Splenic tissues were compressed using the rough surface of two frosted microscope slides and the splenic homogenate obtained was washed with RPMI 1640 medium (LGC Biotecnologia, Cotia, Brazil) and resuspended with syringes and needles of decreasing size (18 G, 22 G and 26 G, respectively), until a homogeneous solution was obtained. Splenic homogenates were centrifuged at 200 g for 10 min and resuspended in 360 μL of filtered distilled water for 10 s in order to promote hemolysis, followed by the addition of 40 μL of 10 × filtered PBS. The centrifugation procedure, followed by the hemolysis procedure, was repeated three times. Splenic samples from each group were plated in pools using 6-well microplates containing 5 ml of RPMI 1640 culture medium (LGC Biotecnologia), supplemented with 0.05 mM β-mercaptoethanol (Sigma-Aldrich), 10% FBS (Cultilab) and 1% penicillin/streptomycin antibiotics (Vitrocell Embriolife). After 2 h of cultivation, the supernatant from each well was collected and transferred to a new plate. The viability of the splenocytes were evaluated using 0.4% trypan blue (Life Technologies) and the splenic samples were kept in an incubator overnight until the co-culture assay.

#### Co-culture assay of tumor cells and splenocytes

For the co-culture assay, MB49 tumor cells labeled with green fluorescent protein (MB49:GFP^+^) were plated in 96-well microplates (5 × 10^4^ cells/well) containing 200 μl of complete DMEM, according to Andrade et al. (2010) [43]. The MB49:GFP^+^ cell culture was kept in an incubator overnight and then used for co-culture with the splenocytes in the proportions of 1:10 and 1:50. This co-cultivation was carried out in quadruplicate and using complete 200 μl of DMEM without phenol red (Thermo Fisher Scientific, Waltham, USA). To determine the co-cultivation cell viability, the co-culture was kept in an incubator and, after 24 h, it was analyzed using the fluorescence system ImageXpress® Micro XLS (Molecular Devices, San Jose, USA), [46]. The cell viability of the co-culture was also analyzed using the resazurin assay as described above.

### Statistical analysis

Quantitative data were analyzed using the one-way or two-way ANOVA statistical and Tukey`s tests using the Prism® software (version 5.0, GraphPad Software, San Diego, USA). The results were expressed as mean ± standard error of the mean (SEM) and, in all analyses, 5% was adopted as the statistical significance limit (*p* < 0.05). In cases where the results did not follow normality, the data were analyzed using the Kruskal–Wallis and Dunn's multiple comparison tests. The survival analysis was performed by the Kaplan Mier method associated with the Gehan-Breslow-Wilcoxon test.

## Data Availability

Not applicable.

## Abbreviations

CAPES: Coordenação de Aperfeiçoamento de Pessoal de Nível Superior
CEUA/UFSCar: Ethics Committee on Animal Use of The Federal University of São Carlos
CFU: Colony-forming unit
CLR: C-type lectin receptor
DMEM: Dulbecco’s modified eagle’s medium
FAPESP: Fundação e Amparo à Pesquisa do Estado de São Paulo
FBS: Fetal bovine serum
IFA: Incomplete Freund’s adjuvant
IVAG: Intravaginal
IVES: Urothelium through intravesical
NAS: Intranasal
RIG-I: Retinoic acid inducible gene-I
SC: Subcutaneous
SEM: Standard error of the mean
STINGs: Stimulator of interferon genes
TLR: Toll-like receptor
TPG: Thermoreversible polymeric gel
TUR: Transurethral resection
UBC: Urothelial bladder cancer
USP-RP: University of São Paulo-Ribeirão Preto
